# Reviewer Acknowledgment

**DOI:** 10.1002/kjm2.12912

**Published:** 2024-11-18

Consistent high‐quality of papers published in “Kaohsiung Journal of Medical Sciences” can only be maintained with the cooperation and dedication of a number of expert referees. The Editors would like to thank all those who have donated the hours necessary to review, evaluate and comment on manuscripts; their conscientious efforts have enabled the journal to maintain its tradition of excellence. We are grateful to the following reviewers for their contributions during 2024.

**B**

Bair Ming‐Jong

**C**

Chan Leong‐Perng

Chan Te‐Fu

Chang Chih‐Peng

Chang Fang‐Rong

Chang Hong‐Yi

Chang Hsueh‐Wei

Chang Jer‐Ming

Chang Kee‐Lung

Chang Kuo‐Wei

Chang Lin‐Li

Chang Long‐Sen

Chang Yo‐Chen

Chen Ruey‐Mei

Chen Yao‐Mei

Chen Chien‐Hung

Chen Chien‐Lin

Chen Chi‐Huang

Chen Chung‐Hwan

Chen Hsiu‐Lin

Chen I‐Chen

Chen Kuan‐Yu

Chen Pai‐Sheng

Chen Shiou‐Lan

Chen Tsan‐Ju

Chen Yen‐Hsu

Chen Yiming

Chen Yuk‐Kwan

Cheng Kuang‐Hung

Cheng Pin‐Nan

Cheng Tsung‐Lin

Chiou Shang‐Jyh

Chiou Tzeon‐Jye

Chiu Chien‐Chih

Cho Shih‐Feng

Chong Yoon‐Bin

Chou Shah‐Hwa

Chou Wen‐Chin

Chu Chih‐Sheng

Chuang Kai‐Jen

Chuang Yao‐Chi

**F**

Fateh Abolfazl

**G**

Gean Po‐Wu

**H**

Ho Yu‐Cheng

Hong Yi‐Ren

Hour Tzyh‐Chyuan

Hsiao George Hsiao

Hsiao Hui‐Hua

Hsieh Tusty‐Jiuan

Hsu Chih‐Yao

Hsu Shih‐Hsien

Hsu Ya‐Ling

Hsu Yao‐Chun

Hsueh Yuan‐Shuo

Huang A‐Mei

Huang Bin

Huang Chih‐Yang

Huang Chung‐Feng

Huang Hung‐Ling

Huang Jee‐Fu

Huang Shang‐En

Huang Shu‐Pin

Huang Yi‐Hsiang

Huang Yu‐Kai

Hung Chao‐Hung

Hung Chi‐Feng

Hung Jen‐Yu

Hung Wen‐Chun

**J**

Ju Da‐Tong

**K**

Kao Shao‐Hsuan

Ko Chih‐Hung

Ko Chih‐Yuan

Ko Ho‐Wen

Kuo Hann‐Chorng

Kuo I‐Ying

Kuo Keng‐Liang

Kuo Yung‐Che

Kuo Yur‐Ren

**L**

Lai Jenn‐Haung

Lai Yu‐Hung

Lan Ming‐Ying

Lee Chien‐Hsing

Lee E‐Jian

Lee Jia‐Jung

Lee Kuo‐Ting

Lee Mei‐Hsuan

Lee Tsong‐Hai

Lee Yen‐Mei

Lee Ying‐Ray

Lee Yung‐Tsai

Lei Wei‐Yi

Li Chia‐Yang

Li Chien‐Feng

Li Shau‐Hsuan

Li Wei‐Ming

Liao Guo‐Shiou

Lin Chi‐Chien

Lin Chih‐Lin

Lin Chih‐Lung

Lin Fan‐Li

Lin Hui‐Ju

Lin Mao‐Shin

Lin Ming‐Hong

Lin Ming‐Yen

Liu Ping‐Yen

Liu Yi‐Chang

Lu Ming‐Ying

Lu Kang

Lue Yi‐Jing

**M**

Ma Hsin‐I

Mo Yin‐Yuan

**P**

Pan Wen‐Chi

**S**

Shiah Shine‐Gwo

Shieh Sheau‐Yann

Su Po‐Lan

Su Tung‐Hung

Sun Hung‐Yu

Sung Chih‐Chien

**T**

Tai Chi‐Ming

Tang Chih‐Hsin

Tsai Eing‐Mei

Tsai Jih‐Jin

Tsai Kuo‐Wang

Tsai Mei‐Ling

Tsai Ming‐Ju

Tsai Tsung‐Ting

Tsai Wen‐Chan

Tsai Yi‐Chun

Tsai Yu‐Chieh

Tseng Yu‐Hsin

Tu Hung‐Pin

**W**

Wang Chih‐Yuan

Wang Jing‐Houng

Wang Shu‐Chi

Wang Wen‐Chen

Wang Wen‐Hung

Wei Yu‐Ju

Wong Ming‐Wun

Wong Wen‐Jang

Wu Chieh‐Hsin

Wu Chin‐Chen

Wu Jeng‐Yih

Wu Wen‐Jeng

**Y**

Yang Chih‐Jen

Yang Chuen‐Mao

Yang Hung‐Chih

Yang Shun‐Fa

Yang Yi‐Sun

Yang Yu‐Chiao

Yeh Jwu‐Lai

Yeh Ming‐Lun

Yeh Yu‐Min

Yen Chia‐Hung

Yen Jeng‐Hsien

Yu Cheng‐Chia

Yu Chia‐Li

Yuan Shyng‐Shiou

